# Dermatopontin in Skeletal Muscle Extracellular Matrix Regulates Myogenesis

**DOI:** 10.3390/cells8040332

**Published:** 2019-04-09

**Authors:** Taeyeon Kim, Khurshid Ahmad, Sibhghatulla Shaikh, Arif Tasleem Jan, Myung-Gi Seo, Eun Ju Lee, Inho Choi

**Affiliations:** 1 Department of Medical Biotechnology, Yeungnam University, Gyeongsan 38541, Korea; xodus3603@naver.com (T.K.); ahmadkhursheed2008@gmail.com (K.A.); sibhghat.88@gmail.com (S.S.); 2 School of Biosciences and Biotechnology, Baba Ghulam Shah Badshah University, Rajouri 185236, India; atasleem@gmail.com; 3 Department of Veterinary Histology, College of Veterinary Medicine, Kyungpook National University, Daegu 702-701, Korea; newmoon93@knu.ac.kr

**Keywords:** dermatopontin, fibromodulin, fibronectin, differentiation, myogenesis, protein-protein interaction

## Abstract

Dermatopontin (DPT) is an extensively distributed non-collagenous component of the extracellular matrix predominantly found in the dermis of the skin, and consequently expressed in several tissues. In this study, we explored the role of DPT in myogenesis and perceived that it enhances the cell adhesion, reduces the cell proliferation and promotes the myoblast differentiation in C2C12 cells. Our results reveal an inhibitory effect with fibronectin (FN) in myoblast differentiation. We also observed that DPT and fibromodulin (FMOD) regulate positively to each other and promote myogenic differentiation. We further predicted the 3D structure of DPT, which is as yet unknown, and validated it using state-of-the-art in silico tools. Furthermore, we explored the in-silico protein-protein interaction between DPT-FMOD, DPT-FN, and FMOD-FN, and perceived that the interaction between FMOD-FN is more robust than DPT-FMOD and DPT-FN. Taken together, our findings have determined the role of DPT at different stages of the myogenic process.

## 1. Introduction

The skeletal muscle comprises 30–50% of the body weight and represents the largest reservoir in the human body [1]. It attaches to the bones and helps in the movement of the skeleton. The skeletal muscle is fundamentally a contractile tissue composed of multinucleated myofibers and stem cells in an inactivated state [2]. The stimulation of the skeletal muscle activates a multipotent precursor cell called muscle satellite cell (MSC), which plays an imperative role in maintaining the functional and structural consistency of the skeletal muscle [3]. MSC affects not only the normal growth of muscles, but also aids in regeneration from scars or disease through a delicate myogenic program. It induces the myogenic cells and maintains a balance between proliferation and differentiation [4,5,6]. Progression of MSCs along the myogenic lineage is initiated with the co-expression of paired box transcription factors (Pax3/Pax7) along with myogenic-regulatory factors (MRFs; including Myf5, MyoD, Mrf4, and myogenin) [7,8,9].

The extracellular matrix (ECM) of the skeletal muscle mainly provides mechanical support and biochemical signals and is usually composed of collagens, laminins, and fibronectin [10]. A number of ECM components are known to play vital roles in the development and maintenance of skeletal muscle [11]. To acquire a deep insight into, and apprehend the physiological mechanism of muscle development, regeneration, and repair, it is essential to study the MSC mechanisms and functions in the surrounding ECM environment [10]. In previous studies, we reported that ECM proteins such as FMOD and matrix gla proteins show significant changes in expression throughout proliferation and differentiation events during myogenesis [12,13,14]. FMOD plays a dynamic role in the regeneration of muscles by increasing the recruitment of MSCs to the sites of injury. It was observed that FMOD bypasses the inhibitory effects of myostatin and maintains its transcriptional activity [14]. Further exploration of the FMOD mechanism through a myogenic program of muscle tissue and MSCs revealed that the dermatopontin (DPT) gene is a hub gene in the network analysis of differentially-expressed genes (DEGs) obtained from microarray data of FMOD knockdown cells. We detected that DPT mediates the expression of myogenic marker genes and participates in myogenesis through the ECM environment [15].

DPT is a widely distributed low molecular weight (22 kDa) tyrosine-rich non-collagenous matrix protein, predominantly expressed in the dermis of the skin and mostly on the surface of collagen fibers [16]. Known functions include binding to the cell surface receptors (integrin α3β1) and mediating adhesion, linking communication between the cell surface of dermal fibroblast and the ECM environment, increasing the transforming growth factor beta 1 (TGFB1) activity, and inhibiting cell proliferation [17,18,19]. DPT is also reported to mediate communication between the ECM environments in the wound healing process via TGFB1, decorin, and fibronectin (FN) [16], and is known to interact with FN, and increase fibril formation and cell adhesion [20].

In the current study, we performed extensive in vitro and in vivo experiments to explore the role of DPT in the regulation of myogenesis. In order to recognize extensively the function of DPT in MSCs, we investigated the association of MSCs with proliferation, adhesion, and differentiation in murine myoblast C2C12 cells. Additionally, we studied DPT and FN in relation to the FMOD mechanism, and the role of DPT in regulating the MSC function during the myogenic program. Finally, we constructed and present a new gene regulation pathway of DPT. Additionally, we predicted the 3D structure of DPT, and performed the in-silico protein-protein interaction (PPI) between DPT-FMOD, DPT-FN, and FMOD-FN. We believe this is the first study that explores the role of DPT in the regulation of skeletal muscle and validates it to be a dynamic component of the skeletal muscle ECM.

## 2. Materials and Methods

### 2.1. Cell Culture

Murine myoblast C2C12 cells (Korean Cell Line Bank, Seoul, Korea) were cultured in DMEM (Dulbecco’s Modified Eagle’s Medium; HyClone, Logan, UT, USA) supplemented with 10% (for cell proliferation) or 2% (for cell differentiation) FBS (fetal bovine serum, Hyclone Laboratories) and 1% P/S (penicillin/streptomycin, Hyclone Laboratories, Logan, UT, USA). At 90–95% cell confluency, the cell culture medium was changed with a differentiation medium, and the media was replaced every 1–2 days.

### 2.2. Gene Knockdown

At 30% cell confluency, DPT, FMOD, Itm2a, FN, COL1α shRNA (1 ng, Santa Cruz Biotechnology, Santa Cruz, CA, USA) and scrambled vector were transfected using transfection reagent (Santa Cruz Biotechnology). The transfected cells were selected with Puromyocin (2 µg/mL, Santa Cruz Biotechnology).

### 2.3. MTT Assay

For attachment and proliferation analyses, DPT_kd_ cells were cultured in proliferation media (10% FBS) and incubated for 1 h or 4 days, respectively. Cells were then washed with PBS and incubated with MTT reagent (0.5 mg/mL; Sigma Aldrich, St. Louis, MO, USA) for 1 h. The generated formazan crystals were dissolved in DMSO (Sigma Aldrich) and the absorbance was measured at 540 nm (Tecan group, Männedorf, Switzerland).

### 2.4. RNA Isolation and qPCR

Total RNAs were extracted from cultured cells using Trizol reagent (Thermo Fisher Scientific, Waltham, MA, USA) as per the manufacturer’s protocol, and then stored at −80°C till further use. Briefly, 2 µg RNA in a cDNA mixture volume of 20 µL was primed with a random hexamer, and subsequently reverse-transcribed using reverse transcriptase (ThermoFisher Scientific, Waltham, MA, USA) as follows: 25 °C for 5 min, 37 °C for 120 min, and 85 °C for 5 min. Real-time PCR was performed for cDNA product (2 μL) and 10 pM of the gene-specific primer, using a 7500-RPM real-time PCR system (Thermo Fisher Scientific) and applying a power SYBR Green PCR Master Mix (Thermo Fisher Scientific) as the fluorescence source. Gene-specific primer sequences are provided in Supplementary Table S1.

### 2.5. Scratch Experiment

DPT_kd_ and DPT_wt_ cells were cultured in the growth medium. At ~95% cell confluency, a scratch was created using a sterile pipette tip, and non-adherent cells were washed out. After scratching, cells were incubated with proliferation medium for 3 days, and the cell recovery (wound closure) was observed by a microscope. Cell recovery was measured from the initial point of the scratched margin to the point of cells recovered, and the recovery rate was measured by calculating the ratio of DTP_kd_ to DPT_wt_.

### 2.6. Western Blot Analysis

Cells were lysed with RIPA buffer containing protease inhibitor cocktail (Thermo Fisher Scientific), and total proteins were quantified using the Bradford assay. Total protein extracts (50 µg) were run on SDS-PAGE (8–12%) and transferred onto PVDF membranes (EMD Millipore, Billerica, MA, USA) using the Bio-Rad mini protein transfer system (Bio-Rad, Hercules, CA, USA). The protein transferred membrane was blocked with 3% skim milk/Tris-buffered saline (TBS) containing Tween 20, for 1 h at room temperature. Blocked membranes were then incubated with the primary antibodies in TBS (DPT, 1:400; FMOD, 1:400; ITM2A, 1:400; COL1α1, 1:400; FN, 1:200; MYOD, 1:400; MYOG, 1:400; MYL2, 1:400; and β-actin, 1:2000), overnight at 4 °C. After washing, the blots were incubated with horseradish peroxidase-conjugated secondary antibodies (goat anti-mouse or anti-rabbit; Santa Cruz Biotechnology) at room temperature for 2 h, and developed using the Super Signal West Pico Chemiluminescent Substrate (Thermo Fisher Scientific).

### 2.7. Immunocytochemistry

Cells were fixed with 4% formaldehyde (Sigma-Aldrich) for 15 min, permeabilized with 0.2% Triton X-100 (Sigma-Aldrich), and subsequently incubated overnight with the primary antibodies [MYOD (1:50), MYOG (1:50), MYL2 (1:50), DPT (1:50), FMOD (1:50), ITM2a (1:50), Col1 alpha 1 FN (1:50), THBS1 (1:50), CYCLIN-A2 (1:50)] at 4 °C in a humid environment. Secondary antibodies (1:100; Alexa Fluor 594 or 488 goat anti-rabbit and goat anti-mouse; Thermo Fisher Scientific) were applied for 1 h at room temperature, after which the cells were counterstained with DAPI (Sigma-Aldrich) and imaged using a fluorescence microscope equipped with a digital camera (Nikon, Melville, NY, USA).

### 2.8. Immunohistochemistry

Expression of Pax7, DPT, and FN in mouse muscle tissue was visualized by immunohistochemistry. Briefly, the paraffin-embedded tissue was deparaffinized, hydrated, and endogenous peroxidase activity was quantified. The sections were blocked with 1% normal goat serum in PBS and incubated with Pax7, DPT and FN antibody (1:50) overnight at 4 °C, followed by incubation with HRP-conjugated secondary antibody (1:100; Santa Cruz Biotechnology). Positive signals were visualized by adding diaminobenzidine and hydrogen peroxide as substrates. The negative control was performed without primary antibody. The stained sections were counter-stained with hematoxylin, dehydrated, mounted, and observed under an optical microscope (Leica, Wetzlar, Germany).

### 2.9. Fusion Index

Fusion index was analyzed as described previously [21]. In brief, cells were fixed with Methanol (MeOH): PBS (1:1), and nuclei were stained with 0.04% Giemsa G250 (Sigma-Aldrich) for 30 min and then washed with PBS, and images were taken randomly at 3 different spots. Additionally, the number of nuclei in myotubes and the total number of nuclei in cells were counted in each field.

### 2.10. Plate Coating with ECM Proteins

For the coating experiments, 50 µg of Type I collagen or 5 µg of FN (Sigma-Aldrich) were added to the plate and incubated for 45 min at room temperature, followed by washing 3 times with PBS.

### 2.11. Animal Experiment

The muscle injury model was prepared as described by Kim et al. [22]. Briefly, mice were anesthetized with avertin (Sigma Aldrich), and 10 mM cardiotoxin (CTX, Sigma Aldrich) was injected into the gastrocnemius muscle. PBS-injected gastrocnemius muscles were used as controls. All experiments were conducted on the 3rd, 7th and 14th day after final injection. Animal samples were collected following a standard protocol approved by the Institutional Animal Care and Use Committee of Yeungnam University (AEC2015-006).

### 2.12. Statistical Analysis

Normalized expression means were compared using Tukey’s Studentized Range to identify significant differences in gene expression. Nominal *p*-values of less than 0.05 are considered statistically significant (SAS Institute, North Carolina, Cary, NC, USA).

### 2.13. 3D Model Generation of DPT

To date, there is no existent 3D structure of DPT in the protein data bank (PDB). Therefore, the sequences of DPT (*Mus musculus*) were obtained from the UniProt database (https://www.uniprot.org/uniprot/Q9QZZ6). A BLASTp search was made against the PDB to find suitable template structures for modeling. Since no significant template was found in the BLASTp search, we performed the template search and automated modeling using threading approaches through various web-servers including I-TASSER, LOMETS, MUSTER, and SPARKS-X. The stereochemical quality of the generated 3D structures was verified using SAVES, VADAR, and ProQ web-servers. The stereochemical properties and Ramachandran plots were then analyzed using Procheck and Rampage, and the validated model was submitted to the Protein Model Data Base (PMDB).

### 2.14. Protein-Protein Interaction

Protein-protein docking interaction was done using PatchDock (https://bioinfo3d.cs.tau.ac.il/PatchDock/); the interaction was further refined and ranked with FireDock (http://bioinfo3d.cs.tau.ac.il/FireDock/). For PatchDock simulations, DPT was set as the receptor, and FN/FMOD was set as a ligand, under default complex-type settings (with clustering RMSD 4.0 Å). For each interaction, 100 predictions were generated using PatchDock, and all predictions were submitted to FireDock to refine the 10 best solutions based on global energy.

## 3. Results

### 3.1. DPT Enhances the Cell Adhesion and Reduces Cell Proliferation

**Figure 1:**
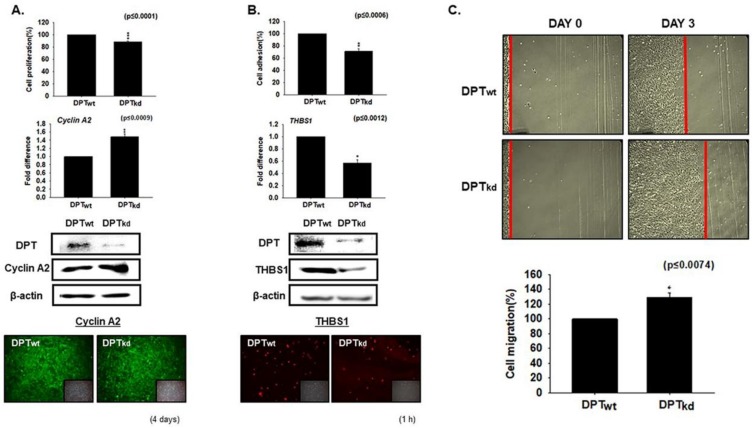
Adhesion and proliferation in dermatopontin (DPT) knockdown cells. (**A**) DPT knockdown (DPT_kd_) and normal cells were cultured in media supplemented with 10% FBS for 4 days. Cell proliferation was evaluated by the MTT assay, mRNA expression by real-time RT-PCR, and proteins expression by Western blot and immunocytochemistry. (**B**) DPT_kd_ and normal cells were cultured with 10% FBS for 1 h. Attachment of cells was measured by MTT assay, mRNA expression by real-time RT-PCR, and proteins expression by Western blot and immunocytochemistry. (**C**) When cells reached ~95% confluency, the monolayer was scratched in normal (DPT_wt_) and DPT_kd_ cells and cultured for 3 more days. The cell recovery ratio was measured by growth distance (red line) from the scratched point to the point of cells recovered. DPT_wt_ indicates cells transfected with the scrambled vector. * *p* ≤ 0.05, ** *p* ≤ 0.001, *** *p* ≤ 0.0001.

To explore the role DPT plays in cell adhesion and proliferation, DPT knockdown (DPT_kd_) and normal cells (DPT_wt_) were cultured in media supplemented with 10% FBS for 4 days (proliferation assay) and 1 h (adhesion assay). A significant decrease in cell proliferation was found in DPT_kd_ cells. However, the expression of Cyclin A2 (a marker gene of the cell cycle) was increased at both the transcriptional (mRNA) and translational (Western blotting and immunocytochemistry) levels in the DPT_kd_ cells (Figure 1A). Further, assessment of cell adhesion by measuring the attachment of cells by MTT assay revealed a decreased rate of adhesion in DPT_kd_ cells. THBS1 is an adhesive ECM protein known to interact with major components of ECM (collagen V, fibronectin, laminin, integrin αvβ1) [23]. Consistent with the above results, a significant decrease was observed for THBS1 expression in the DPT_kd_ cells, at both the mRNA and protein levels (Figure 1B). We next measured the cell migration rate (proliferation) by performing the scratch experiment, wherein a scratch was created in a ~95% confluent monolayer of the cultured DPT_wt_ and DPT_kd_ cells. The cell number at the start point in both (DPT_wt_ and DPT_kd_) plates were the same and the growth rate was observed on the third day. The cell migration rate was found to be pronounced in DPT_kd_ cells as compared to the DPT_wt_ cells (Figure 1C). Altogether, our results suggest that DPT enhances cell adhesion and reduces cell proliferation during the course of myogenesis in C2C12 cells.

### 3.2. DPT Expression during Myoblast Differentiation

**Figure 2:**
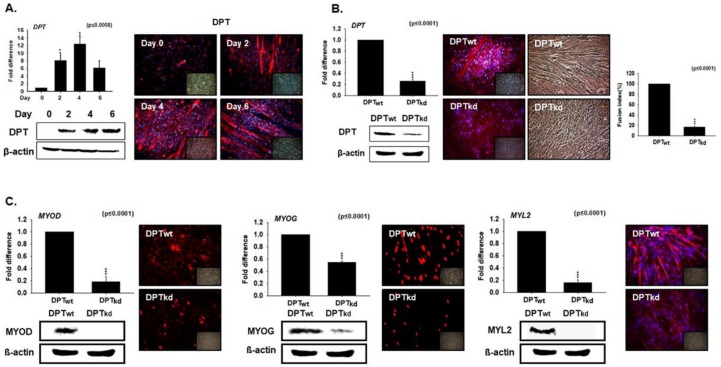
The effect of switching cells from proliferation to differentiation on DPT expression, and DPT expression during myoblast differentiation. (**A**) C2C12 cells were cultured with 2% FBS for 0, 2, 4, and 6 days. The relative DPT mRNA level was assessed by real-time RT-PCR, and protein expression was evaluated by Western blot and immunocytochemistry. (**B**) DPT knock-down was performed and cells were cultured with 2% FBS for 4 days. Myotube formation and fusion index were evaluated by Giemsa staining, DPT mRNA expression by real-time RT-PCR, and protein expression by Western blot and immunocytochemistry. (**C**) mRNA expression by real-time RT-PCR and protein expression by Western blot and immunocytochemistry in DPT_kd_ and DPT_wt_ cells. DPT_wt_ indicates cells transfected with the scrambled vector. * *p* ≤ 0.05, ** *p* ≤ 0.001, *** *p* ≤ 0.0001.

To elucidate the involvement of DPT during myogenesis, we performed a time point study of DPT in differentiating C2C12 cells. Expression of DPT at both mRNA and protein levels showed a progressive increase during the transition from Day 0 (proliferation) to Day 4 (differentiation), with a small decline (mRNA) at Day 6 (Figure 2A). Next, the DPT_kd_ cells were incubated in differentiation media for 4 days. Myotube formation, mRNA and protein levels of DPT were significantly decreased in DPT_kd_ relative to the DPT_wt_ cells (Figure 2B). Furthermore, expressions of the myogenic marker genes (MYOD, MYOG, and MYL2) were significantly decreased in the DPT_kd_ cells, both at the transcriptional and translational levels (Figure 2C). These findings suggest the active role of DPT during myogenic differentiation.

### 3.3. Knockdown Effect of FN during Myoblast Differentiation

**Figure 3:**
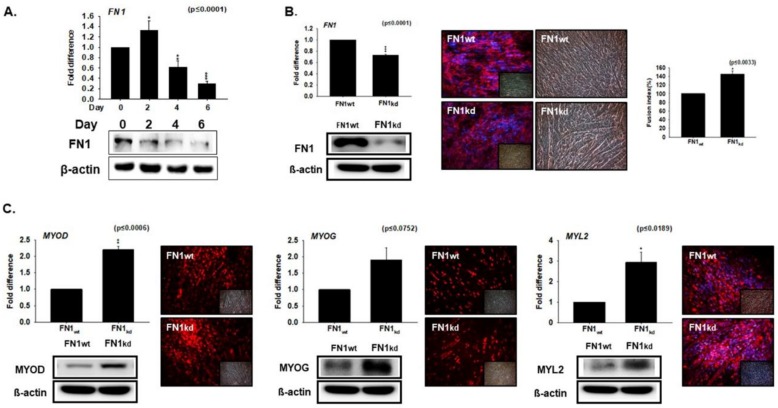
Fibronectin (FN) knockdown expression during myoblast differentiation. (**A**) C2C12 cells were cultured with 2% FBS for 0, 2, 4, and 6 days. FN mRNA levels were assessed by real-time RT-PCR, and protein expression by Western blot. (**B**) FN knock-down was performed and cultured with 2% FBS for 4 days. mRNA expression were assessed by real-time RT-PCR, protein expression by Western blot and immunocytochemistry, And myotube formation and fusion index by Giemsa staining. (**C**) The mRNA expression assessed by real-time RT-PCR and protein expression by Western blot and immunocytochemistry in FN_kd_ and FN_wt_ cells are shown. FN_wt_ indicates cells transfected with the scrambled vector. * *p* ≤ 0.05, ** *p* ≤ 0.001, *** *p* ≤ 0.0001.

The expression of FN1 was evaluated in the C2C12 myoblast cells. Cells were cultured in the desired media for 0, 2, 4 or 6 days. A slight increase in levels was observed from Day 0–Day 2, subsequent to a progressive decrease in the FN1 expression during cell transition from Day 2 (proliferation) to Day 4 and Day 6 (differentiation) (Figure 3A). To investigate the role of FN1 in muscle differentiation, the FN1 was knocked-down in C2C12 cells (FN_kd_ cells). After culturing in differentiation media for 4 days, the mRNA and protein expression of FN1 was found to be significantly reduced in the FN_kd_ cells, relative to the FN_wt_ cells (Figure 3B). An increase in the fusion indices observed in FN1_kd_ cells reflects its role with respect to regulating the differentiation process (Figure 3B). Consistent with this, a significant increase in the expressions of myogenic factors (MYOD, MYOG, and MYL2) were observed in the FN1_kd_ cells (Figure 3C). Taken together, findings from the results presented in Figure 2 and Figure 3 suggest that DPT and FN1 represent opposing effects in the expression of myogenic markers genes.

### 3.4. Interaction of DPT with FN and FMOD during Differentiation

**Figure 4:**
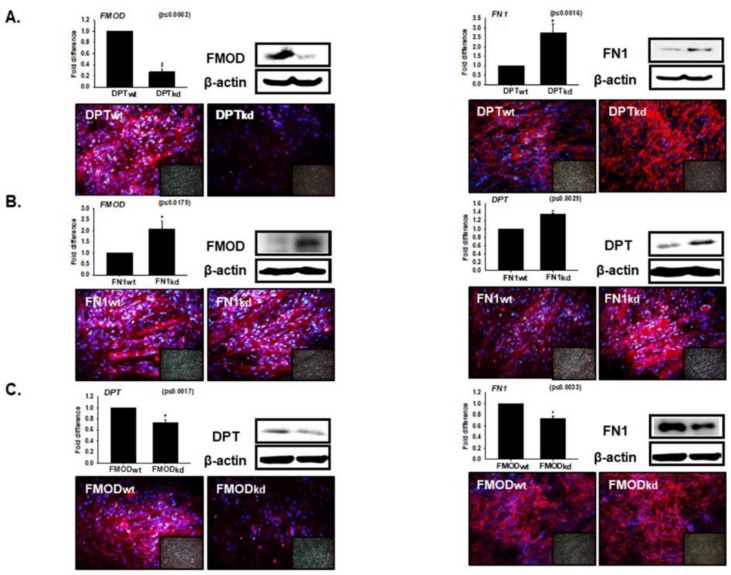
Relationship of DPT with fibromodulin (FMOD) and FN during myoblast differentiation. Knockdown cells of FMOD, DPT, and FN were incubated with 2% FBS for 4 days. (**A**) FMOD and FN mRNA expression were evaluated by real-time RT-PCR, and protein expression by Western blot and immunocytochemistry in DPT_kd_ cells. (**B**) FMOD and DPT mRNA expression was assessed by real-time RT-PCR, and protein expression by Western blot and immunocytochemistry in FN_kd_ cells. (**C**) DPT and FN mRNA expression were assessed by real-time RT-PCR, and protein expression by Western blot and immunocytochemistry in FMOD_kd_ cells. DPT_wt_, FMOD_wt_, and FN_wt_ indicate cells transfected with the scrambled vector. * *p* ≤ 0.05, ** *p* ≤ 0.001, *** *p* ≤ 0.0001.

To investigate the expression of FN1 and FMOD in DPT_kd_ and vice-versa, knockdowns of FMOD and FN1 were performed in C2C12 cells. On incubating the DPT_kd_ and DPT_wt_ cells in differentiation media for 4 days, a significant increase was observed in the expression (mRNA and protein) of FN1 and a decrease in FMOD level (Figure 4A). Evaluation of the expression levels of DPT, FN1, and FMOD in FN1_kd_ and FMOD_kd_ cells revealed a significant increase in FMOD and DPT expression in FN1_kd_ cells (Figure 4B). Additionally, a significant decrease was obtained in the expression levels of DPT and FN1 in FMOD_kd_ cells, both at the mRNA and protein levels (Figure 4C). These findings further support the results that show the contrasting effect of FN with DPT (Figure 2 and Figure 3).

### 3.5. Compensation Effect of Fibronectin with DPT

**Figure 5:**
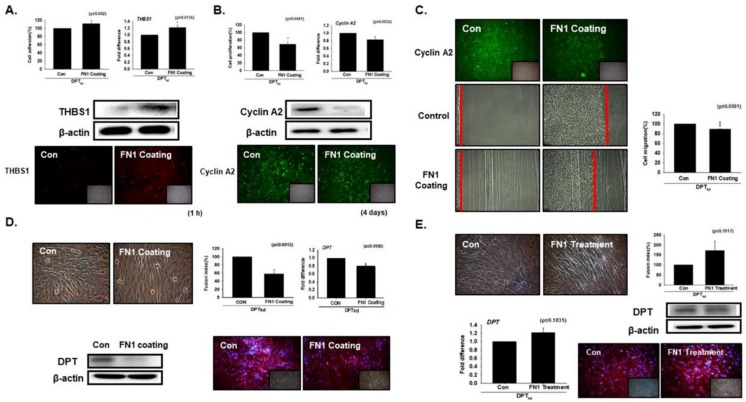
Compensation effect of fibronectin with DPT knockdown. (**A**) DPT knock-down was performed and cells were cultured in 10% FBS with or without the FN-coated plate for 1 h. Cell adhesion or cell attachment was evaluated by MTT assay, THBS1 mRNA expression by real-time RT-PCR, and protein expression by Western blot and immunocytochemistry. (**B**) DPT_kd_ cells were cultured in 10% FBS with or without FN-coated plate for 4 days. Cell proliferation was evaluated by MTT assay, Cyclin A2 mRNA expression by real-time RT-PCR, and protein expression by Western blot and immunocytochemistry. (**C**) Scratch assay was performed when cells reached a confluency of ~95%; cells were scratched in both plate (with and without FN-coated) and incubated for 3 more days. Differences between the migration pattern of FN1 coated plate and non-coated (control) plate. Expression of Cyclin A2 protein were assessed by immunocytochemistry. (**D**) DPT_kd_ cells were cultured with 2% FBS in 1mM FN-coated plate for 4 days. Myotube formation and fusion index were assessed by Giemsa staining, DPT mRNA expression by real-time RT-PCR, and protein expression by Western blot. (**E**) DPT_kd_ cells were cultured in 2% FBS supplemented with 1 mM FN protein for 4 days. Myotube formation and fusion index were assessed by Giemsa staining, DPT mRNA expression by real-time RT-PCR, and protein expression by Western blot. * *p* ≤ 0.05, ** *p* ≤ 0.001, *** *p* ≤ 0.0001.

To determine the compensatory effect of FN1 with DPT, DPT_kd_ cells were cultured in 10% FBS with or without FN1 coating for 1 h. Enhanced cell adhesion with increased THBS1 gene expression was found in DPT_kd_ cells cultured in FN1 coated plates, relative to the non-coated plates (Figure 5A). Likewise, DPT_kd_ cells were cultured for 4 days in FN1 coated plate for proliferative analysis. Compared to cell adhesion, a decreasing trend in cell proliferation with reduced Cyclin A2 expression was found in DPT_kd_ cells cultured in FN1 coated plates (Figure 5B). Additionally, decrease in the cell migration rate with reduced Cyclin A2 expression, and reduced myotube formation with decreased DPT mRNA and protein expressions were observed in DPT_kd_ cells cultured in FN1 coated plates (Figure 5C,D). Interestingly, increases in the myotube formation with increased DPT mRNA and protein expressions were observed in DPT_kd_ cells supplemented with FN1 (Figure 5E). Here, we postulate the robust compensatory effect between FN1 and DPT, which highlights the importance of their expression with respect to regulation in the myogenic program.

### 3.6. DPT and FN1 in Muscle Regeneration

**Figure 6:**
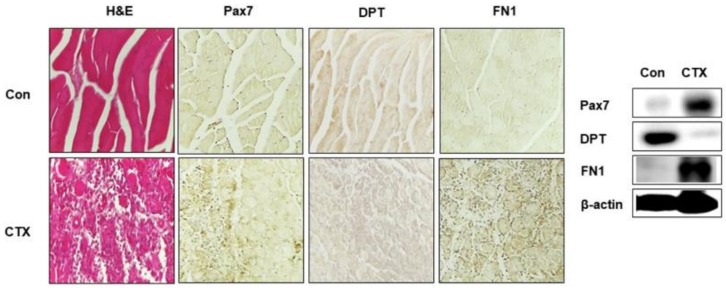
Muscle regeneration analysis. Cardiotoxin (CTX) was injected in the gastrocnemius muscle of mice and maintained for 3 days. The muscle tissue was collected, and morphology was observed by H & E staining. Expressions of Pax7 (control), DPT, and FN proteins by immunohistochemistry and Western blot are shown.

To demonstrate the role of DPT and FN1 proteins in the regeneration program, CTX was injected in the gastrocnemius muscle of mice and maintained for 3 days. Expression of Pax7 (used as a control), DPT and FN1 protein were analyzed by immunohistochemistry and Western blotting. Following CTX injection, the myofibers degrade, with corresponding increases in Pax7 and FN1 expression. However, DPT expression was found to decrease in the muscle injury model after CTX administration (Figure 6). Interestingly, an increase in FN1 expression supports the results obtained in our in vitro experiments (Figure 2 and Figure 3). Additionally, DPT expression was increased in Day 7 of CTX injection during the differentiation phase (Supplementary Figure S1). Therefore, DPT expression is crucial during muscle regeneration.

### 3.7. 3D Protein Modeling of DPT

**Figure 7:**
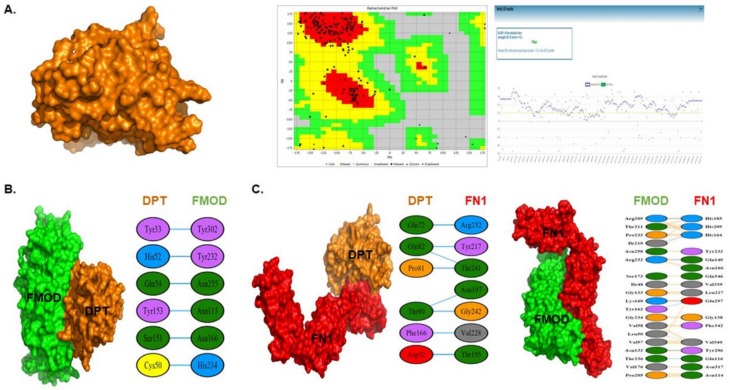
In silico structure prediction and validation of DPT. (**A**) A 3D structure of DPT was predicted by protein modeling servers (e.g., SPARKX) and validated by in silico tools and web-servers. Ramachandran plot analysis of the residues present in DPT and validation of modeled structure by Verify 3D web-server are shown. (**B**) The protein-protein interaction performed by PatchDock and FireDock web servers, and schematic 2-D representations of protein-protein complexes by Ligplot for DPT-FMOD. (**C**) DPT-FN and FMOD-FN.

Automated protein modeling using threading approaches were carried out through various web-servers. The model generated by SPARKS-X showed the best validation (Figure 7A). The Ramachandran plot analysis of the modeled structure showed that 87.4% of residues lie in the favored region (Figure 7A). The accuracy of the 3D model was also confirmed by VERIFY 3D as 84.08% of residues showed a score higher than 0.2. Furthermore, the validation by VADAR web-server, which analyses the different parameters (viz. ‘fractional accessible surface area’, ‘3D profile quality index’, and ‘stereo/packing quality index’) revealed that the residues in the 3D modeled structure were within the favorable range. The protein quality prediction by ProQ web-server revealed the ‘Predicted LG Score’ and ‘Predicted MaxSub’ as 2.419 and 0.050, respectively, thereby confirming the obtained 3D structure as a good model. The validated 3D structure has been deposited to the PMDB (ID: PM0081951), a database which collects 3D protein models obtained by structure prediction methods.

### 3.8. Protein-Protein Interaction

Protein-protein interaction (PPI) between DPT-FMOD, DPT-FN1, and FMOD-FN1 explored the binding efficacy of these proteins in terms of global energy. The global energy for ‘DPT-FMOD’, ‘DPT-FN1’ and ‘FMOD-FN1’ interactions predicted by the FireDock server were found to be −41.66, −32.73, and −60.09 kcal/mol, respectively. The interacting amino acid residues in these PPI reveals that the number of hydrogen bonds and hydrophobic interactions are maximum in the FMOD-FN1 interaction with the highest binding energy, while the interaction of DPT with FMOD and FN1 is not as robust (Figure 7B,C).

## 4. Discussion

In previous studies, we explored the role of FMOD in myogenesis as it mainly controls several genes related to the process. The contribution of the FMOD gene in wound healing and in the assembly of ECM components (e.g., collagen) is well documented in earlier studies [24,25,26]. FMOD mediates the expression of myogenic marker genes and participates in myogenesis through the ECM environment. It also functions as an upstream gene, controlling the integral membrane protein 2A (Itm2a) and collagen 1α1 during the differentiation process. DPT was identified as a hub gene in the network analysis of DEGs of FMOD knockdown cells using microarray analysis [15]. In continuation of our previous work, the present study was undertaken to determine extensively the role of DPT in myogenesis. In order to understand the function of DPT in the regulation of myogenesis, we investigated the association of MSCs with proliferation, adhesion, and differentiation in the mouse C2C12 myoblast cell line. Additionally, we deliberated the expression of DPT and FN1 in relation to the FMOD mechanism and found that DPT regulates MSC function during the myogenic program. Additionally, we constructed a new gene regulation pathway of DPT, which reveals the association between DPT, FN1, and FMOD in the milieu of myogenesis.

DPT is expressed in various tissues; a known function includes binding to cell surface receptors, thereby arbitrating cell adhesion and decreasing cell proliferation in various tissues [16,17,27,28]. Earlier studies report that DPT regulates the ECM environment by triggering the fibrillogenesis of collagen and FN1, and regulates the interaction between decorin and TGFB1 [29]. Since DPT promotes cell adhesion and is involved in ECM assembly, it maintains the ability to regulate various physiological processes. Down-regulation of DPT may be allied with uterine leiomyomas, systemic sclerosis, cutaneous fibrosis, and numerous cancers [30,31,32]. Recently, Guo et al. reported that overexpression of DPT hinders the proliferation of papillary thyroid cancer (PTC), both in vivo and in vitro. Additionally, they found that DPT regulates CDK4, CDK6, and p21 via ‘MEK-ERK-MYC’ signaling to suppress the PTC proliferation [33].

We performed a series of in vitro experiments. The cell adhesion and proliferation assay revealed that DPT enhances the cell adhesion whereas it decreases the cell proliferation in C2C12 cells (Figure 1). The significantly decreased mRNA and protein expression levels of THBS1 in DPT_kd_ cells prove that DPT promotes cell adhesion in myogenesis. THBS1 is a marker for cell adhesive ECM protein that interacts with major structural components of ECM [34] (Figure 1B). The scratch assay measures cell migration (proliferation); our results showed faster cell migration rate in DPT_kd_ cells as compared to DPT_wt_ cells, demonstrating that DPT inhibits the cell proliferation in myogenesis. This was further confirmed by the elevated expression of Cyclin A2, a marker gene of the cell cycle.

The expression of DPT during myoblast differentiation signifies an active role in the myogenic differentiation process. In DPT_kd_ cells, the decreased myotube formation and fusion indices, as well as reduced expression of MYOD, MYOG, and MYL2 (Figure 2), confirms the active role of DPT as a regulatory protein in the myogenic process. In contrast, significantly increased expressions of myogenic factors were observed in the FN_kd_ cells during myoblast differentiation (Figure 3). These results provide evidence that DPT promotes differentiation while FN down-regulates it. 

The expression of DPT in FN1 and FMOD (and vice versa) explores the inter-relationship between these ECM genes, which are known to be actively involved in the myogenic program (Figure 4A–C). We observed that DPT and FN1 negatively regulate each other (as seen in DPT_kd_ cells), the expression of FN1 is significantly increased (Figure 4A), and the expression of DPT was found to be significantly higher in FN1_kd_ cells (Figure 4B). DPT and FMOD positively regulate each other (Figure 4A,C), while FMOD regulates positively to FN1 (Figure 3C) but FN1 shows negative regulation to FMOD (Figure 4B) during myogenesis.

Previous studies have reported that DPT interacts with FN1 and promotes the formation of insoluble FN1 fibrils (activated FN1) [20]. FN1 is a well-studied ECM protein abundantly found in myoblasts and evidently it decreases in the differentiation of myotubes [35]. FN1 binds to the laminin and collagens and contributes to adhesion, migration, and differentiation of myoblasts [36]. It is also found to be involved in the expansion of MSCs via Wnt7a signaling [37,38]. In the current study, we investigated the strong compensatory effect of both DPT and FN1 in the microenvironment of skeletal muscle, which demonstrates the importance of DPT in myogenesis. Although there was a sufficient compensatory effect in attachment and proliferation, we observed reduced differentiation and decreased expression of DPT in the FN1 coated plate. Therefore, the data for the contradictory expression patterns of DPT and FN1 (Figure 4A,B) are additional proofs. DPT expression was decreased at Day 3 (proliferation stage) of CTX injection and increased at Day 7 (differentiation stage) of CTX injection compared to the control in the in vivo experiments. These results provide strong evidence of the role of DPT in muscle regeneration processes (Figure 6, Supplementary Figure S1).

Since the 3D structural information (X-ray crystal structure) of DPT is unavailable, we first undertook the effort to generate an in-silico 3D structure using state of art in-silico tools. We successfully developed a hypothetical 3D structure and validated it using online validation tools (Figure 7A), which can now be accessed via the PMDB repository (ID: PM0081951). After generating the 3D structure of DPT, we performed the PPI amongst DPT, FN1, and FMOD to explore their interaction efficacies. Protein-protein interaction was performed using the PatchDock and FireDock web servers, and the binding efficacy is presented in terms of global energy. The global energy of DPT-FN interaction was found to be the minimum (−32.73) as compared to DPT-FMOD (−41.66) and FMOD-FN1 (−60.09). The in-depth amino acid residue interaction calculated by Ligplot reveals a strong hydrophobic interaction and hydrogen bonding in FMOD-FN1, as compared to DPT-FN1 and DPT-FMOD (Figure 7B).

**Figure 8:**
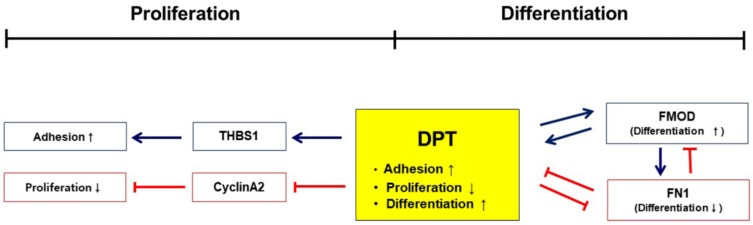
Schematic representation of DPT effects in myogenesis. A representation of the diverse role of DPT in myogenesis is shown. DPT enhances the cell adhesion by inducing THBS1, and decreases cell proliferation by inhibiting Cyclin A2. A demonstration of the interrelationship between DPT, FMOD, and FN is also shown.

Summarizing the outcomes of this study, we constructed a scheme (Figure 8) which visibly demonstrates that: (1) DPT is actively involved in the myogenic program as it increases cell adhesion, decreases cell proliferation, and enhances differentiation; (2) DPT and FN1 show inhibitory effects to each other in the myogenic milieu; and (3) DPT and FMOD positively regulate each other and enhance muscle differentiation.

## Supplementary Materials

The Supplementary Materials are available online at https://www.mdpi.com/2073-4409/8/4/332/s1.
