# Supplementary material for: Dermatopontin in Skeletal Muscle Extracellular Matrix Regulates Myogenesis
Source: Cells. 2019 Apr 9;8(4):332. doi: 10.3390/cells8040332 (PMC6523808; doi:10.3390/cells8040332)

Supplementary Table 1. PCR primers

Species	Gene name	Product size (bp)	Tm	Sequence (F)	Sequence (R)
Mouse	MyoD	213	59	5'-aggagcacgcacacttctct-3'	5'-tctcgaaggcctcattcact-3'
	Myogenin	185	59	5'-tccagtacattgagcgccta-3'	5'-caaatgatctcctgggttgg-3'
	MYL2	177	59	5'-aaagaggctccaggtccaat-3'	5'-cctctctgcttgtgtggtca-3'
	Cyclin A2	227	56	5'-ctgtctctttacccggagca-3'	5'-agtgatgtctggctgcctct-3'
	Thbs1	162	59	5'-catgtggcaatggaattcag-3'	5'-aacaggacgaccatggagac-3'
	FMOD	155	59	5'-tgcagaagatccctcctgtc-3'	5'-cttgatctcgttcccatcca-3'
	DPT	224	54	5’-ggatcgtgagtggcaatttt-3’	5’-cgaattcgcagtcgtagtca-3’
	FN1	241	59	5'-aaggacaaccgaggaaacct-3'	5'-gcttgtttccttgcgacttc-3'

Supplementary Table 2. shRNA sequence information

Gene name	Catalog number	Hairpin sequence	Corresponding shRNA
**DPT shRNA Plasmid (m) is a pool of 3 different shRNA plasmids**			
**DPT**	sc-143012-SHA	5’-gatccctgacagacaatggaactattcaagagatagttccattgtctgtcagttttt-3’	Sense: 5’-cugacagacaauggaacuatt-3’
			Antisense: 5’-aguuccauugucugucagtt-3’
	sc-143012-SHB	5’-gatcccagtggaagttcatcatgtttcaagagaacatgatgaacttccactgttttt-3’	Sense: 5’-caguggaaguucaucaugutt-3’
			Antisense: 5’-caugaugaacuuccacugtt-3’
	sc-143012-SHC	5’-gatccctgtgattcgcactataacttcaagagagttatagtgcgaatcacagttttt-3’	Sense: 5’-ugugauucgcacuauaactt-3’
			Antisense: 5’-guuauagugcgaaucacagtt-3’
**FN1 shRNA Plasmid (m) is a pool of 3 different shRNA plasmids**			
**FN1**	sc-35371-SH	5’- gatcccaaatctcctgcctgggacttcaagagagtcccaggcaggagatttgttttt -3	Sense: 5’- caaaucuccugccugggactt -3’
			Antisense: 5’- gucccaggcaggagauuugtt -3’
**FMOD shRNA Plasmid (m) is a pool of 3 different shRNA plasmids**			
**FMOD**	sc-44823-SHA	5’- gatccctacatggcaaccagattattcaagagataatctggttgccatgtagttttt -3	Sense: 5’- cuacauggcaaccagauuatt -3’
			Antisense: 5’- uaaucugguugccauguagtt -3’
	sc-44823-SHB	5’- gatccctactagacctgagttatattcaagagatataactcaggtctagtagttttt -3	Sense:5’- cuacuagaccugaguuauatt -3’
			Antisense:5’- uauaacucaggucuaguagtt -3’
	sc-44823-SHC	5’- gatccgcaacaggatcaatgagttttcaagagaaactcattgatcctgttgcttttt -3	Sense:5’- gcaacaggaucaaugaguutt -3’
			Antisense: 5’- aacucauugauccuguugctt -3’

Supplementary Figure 1

Supplementary figure1.

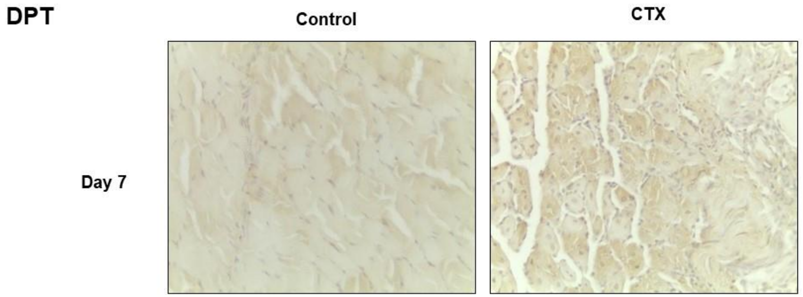

Supplement: Supplementary file 1 [file cells-08-00332-s001.pdf]
